# Cerebrovascular Variants and the Role of the Selfish Brain in Young-Onset Hypertension

**DOI:** 10.1161/HYPERTENSIONAHA.121.18612

**Published:** 2022-03-16

**Authors:** Nathan E. Manghat, Elizabeth Robinson, Konstantina Mitrousi, Jonathan C.L. Rodrigues, Thomas Hinton, Julian F.R. Paton, Richard G. Wise, Angus K. Nightingale, Emma C. Hart

**Affiliations:** Department of Radiology, Bristol Royal Infirmary, University Hospitals Bristol and Weston NHS Foundation Trust, United Kingdom (N.E.M., E.R., K.M.).; Cardionomics Research Group, School of Physiology, Pharmacology and Neurosciences, University of Bristol, United Kingdom (N.E.M., K.M., T.H., A.K.N., E.C.H.).; Department of Radiology, Royal United Hospitals Bath NHS Foundation Trust (J.C.L.R.).; Department of Health, University of Bath, United Kingdom (J.C.L.R.).; Manaaki Manawa, The Centre for Heart Research, Department of Physiology, Faculty of Medical & Health Sciences, University of Auckland, New Zealand (J.F.R.P.).; Cardiff University Brain Research Imaging Centre, School of Psychology, Cardiff University (R.G.W.).; Department of Neuroscience, Imaging and Clinical Sciences (R.G.W.), “G. D’Annunzio” University of Chieti-Pescara, Italy.; ITAB-Institute for Advanced Biomedical Technologies (R.G.W.), “G. D’Annunzio” University of Chieti-Pescara, Italy.; Clinical Research Facility, University Hospitals Bristol and Weston NHS Foundation Trust, United Kingdom (A.K.N., E.C.H.).

**Keywords:** blood pressure, circle of Willis, hypertension, young adults, vertebral artery

## Abstract

Variants in the posterior anatomy of the cerebral circulation are associated with hypertension and lower cerebral blood flow in midlife (age ≈55 years); however, whether these variants are a result of aging or long-term exposure to high blood pressure is unclear. Additionally, the role these variants play in early onset of hypertension (<40 years) and poor cerebral perfusion in this population is unknown.

**Methods:**

We retrospectively examined whether specific cerebrovascular variants (vertebral artery hypoplasia and absent/hypoplastic posterior communicating arteries (an incomplete posterior circle of Willis) measured via magnetic resonance angiography) were associated with a diagnosis of hypertension in 220 young adults (<40 years; n=164 primary hypertensive [mean age±SD, 32±6 years] and n=56 [30±6 years] normotensive adults). Whether cerebrovascular variants were associated with lower cerebral blood flow (phase-contrast angiography) was measured in the hypertensive group only (n=146).

**Results:**

Binary logistic regression (adjusted for age, sex, and body mass index) showed that vertebral artery hypoplasia with an incomplete posterior circle of Willis was associated with hypertension diagnosis (*P*<0.001, odds ratio; 11.79 [95% CI, 3.34–41.58]). Vertebral artery hypoplasia plus an incomplete circle of Willis was associated with lower cerebral blood flow in young adults with hypertension (*P*=0.0172).

**Conclusions:**

Vertebral artery hypoplasia plus an incomplete posterior circle of Willis independently predicts hypertension in young adults suggesting that this variant is not acquired with aging into midlife. Importantly this variant combination was associated with lower cerebral perfusion, which may have long-term consequences on cerebrovascular health in young adults with hypertension.

**Novelty and Relevance**

**What Is New?**

- We show that hypoplasia of the vertebral arteries combined with absent or hypoplastic posterior communicating arteries is independently associated with hypertension diagnosis in young adults (<40 years). These variants are typically thought to be congenital suggesting that these variants could be causal in the pathophysiology of hypertension, but more work is needed.

**What Is Relevant?**

- Variants in the posterior circulation were associated with lower cerebral blood flow in patients with young-onset hypertension, despite 70% being prescribed antihypertensive therapy.

**Clinical/Pathophysiological Implications?**

This is the first data to show that variants in the posterior circulation may have an impact on cerebral blood flow in younger adults with hypertension, which could have long-term consequences on cerebrovascular health with aging.

Hypertension is the leading contributor to the global burden of disease and mortality.^1^ In the United Kingdom, ≈27% of adults have hypertension and the number of young adults with hypertension is growing. In 2016, the Health Survey for England showed that 17.8% of men and 11.8% of women 35 to 44 years of age had hypertension. Worldwide prevalence estimates among adults aged 20 to 29 years and 30 to 39 years stand at 10.1% and 15.5%, respectively.^2^ Alarmingly, people who develop hypertension in young adulthood (<40 years) are more likely to have earlier onset of coronary heart disease, heart failure, stroke, transient ischemic attacks, and mild cognitive impairment.^3^

Despite the high prevalence of hypertension, the underlying causes of hypertension are not fully understood. Mechanisms contributing to elevated blood pressure (BP) include activation of the renin-angiotensin-aldosterone and sympathetic nervous systems,^4^ but the specific triggering mechanisms are unclear. Animal models of high BP indicate that hypertension may develop as a compensatory mechanism in order to sustain adequate cerebral perfusion.^5–7^ Cates et al^6^ showed that in spontaneously hypertensive rats, vertebrobasilar remodeling, and the development of low cerebral blood flow (CBF) occurred before the onset of hypertension, suggestive of a causal association.

We have previously found a higher prevalence of cerebrovascular variants, including incomplete posterior circle of Willis (ipCoW) and vertebral artery hypoplasia (VAH) in people with midlife hypertension (>50 years).^8,9^ These variants are associated with diminished CBF and increased cerebrovascular resistance in hypertensives compared to age-matched controls.^4,5^ Warnert et al^8^ showed that cerebrovascular resistance was increased before the increase in sympathetic nerve activity and onset of hypertension in a cross-section of participants with groups of different levels of BP. This was consistent with the finding that cerebrovascular resistance was a stronger predictor of hypertension than body mass index (BMI) and age. Taken together the findings suggest that cerebral hypoperfusion and elevated cerebrovascular resistance may precede the activation of the sympathetic nervous system; this is known as the selfish brain hypothesis of hypertension.^6^

However, supporting data in humans is from cross-sectional studies in middle-aged cohorts.^8^ Whether variants in the posterior cerebral circulation are associated with primary hypertension and hypoperfusion in young adults (<40 years) is not known. Variants such as VAH are thought to be congenital or a consequence of early life developmental events suggesting that these observed variants are present before the onset of hypertension; however, there is no data to support this. An alternative explanation is that the observed hypoplasia is a result of aging and long-term exposure to high BP. We hypothesize that variants in the anatomy of the posterior cerebral circulation and circle of Willis are a cause of primary hypertension rather than a consequence of it. Thus, if variants in posterior cerebral arteries are causal to hypertension, then in people diagnosed with young-onset hypertension who have less time exposure to higher BPs, the prevalence of posterior circulation variants should be higher versus age-matched controls.

Therefore, we examined whether the incidence of cerebrovascular variants was different between patients diagnosed with young-onset (primary) hypertension and normotensive controls. We also examined whether posterior variants were linked to reduced CBF in patients with young-onset hypertension.

## Methods

The data that support the findings of this study are available from the corresponding author upon reasonable request.

### Study Design

This was a retrospective cross-sectional study, where patients diagnosed with early-onset hypertension (≤40 years) were identified and matched with control (normotensive) participants. Data were obtained from 3 sources: (1) the hypertensive group were identified from patients attending our group’s tertiary care hypertension clinic, (2) consecutive normotensive individuals attending for brain and cerebrovascular magnetic resonance imaging (MRI) scans as part of usual clinical investigation for pulsatile tinnitus with no causative findings were identified to be healthy controls, and (3) normotensive participants volunteering for a previous study (controls; 11/SW/0207). The local Research Ethics committee confirmed that the study conformed to the governance arrangements for research ethics committees.

### Study Population

A total of 173 young patients (≤40 years) with primary hypertension referred to the Bristol Heart Institute tertiary hypertension clinic between February 2015 and April 2021 were identified for analyses (secondary causes of hypertension had been excluded in clinic and by our previously described institutional comprehensive hypertension clinic cardiovascular MRI protocol).^10^ Patients attending the clinic were referred from Primary Care based on National Institute Institution for Health and Care Excellence guidelines. Cases included were from consecutive referrals by the hypertension clinic to the Cardiovascular Magnetic Resonance Unit in the National Institute for Health Research Bristol Cardiovascular Biomedical Research Centre in the Bristol Heart Institute. Exclusion criteria were patients with hypertrophic cardiomyopathies, previously repaired coarctation of the aorta, no MR angiogram of the cerebral circulation, or incomplete imaging of all segments of the vertebral arteries (Figure S1). We excluded 9 cases; thus, the hypertension sample size was n=164. Control participants were identified from 2 cohorts. First, 58 normotensive patients (≤40 years) attending MRI at the Bristol Royal Infirmary for MR cerebral angiography indicated for pulsatile tinnitus were identified (November 2018–November 2019). Cases were excluded if there was diagnosis of arteriovenous malformations or aneurysm, history of congenital heart disease, lack of cerebral MRA or incomplete MRA of the vertebral arteries, taking antihypertensive medication, and lack of BP data to confirm normotension status (clinic BP <140/90 mm Hg; Figure S1). Based on this criteria, we excluded 29 cases from the 58 identified; thus 29 cases were used from this set of cases. Second, 27 normotensive control participants volunteering in a previous MRI study were included (inclusion criteria <40 years).^8^ Total control sample size was 56. Table S1 shows patient characteristics.

### BP Measurements

Average systolic and diastolic BP were measured from both arms after rest, using standard automated sphygmomanometery with an appropriately sized cuff.^11^ In a subgroup of hypertensive patients (n=84) and normotensive participants (n=27), 24-hour ambulatory BP monitoring was completed^11^ (Table S1).

### MRI Procedures

Three-dimensional time-of-flight angiography was used to measure arterial anatomy and was completed on 2 different MRI scanners. First, in all the hypertensive and 29 normotensive subjects images were acquired during free breathing at 1.5T (Siemens Avanto Magnetom, Siemens Healthineers; echo time/repetition time: 7/23 ms, flip angle 25 degrees, phase encoding R/L, field of view: 200 mm, phase field of view 94%, matrix: 256×94 pixels, slice resolution 85%, slice thickness: 0.8 mm, voxel dimensions 0.8×0.8×0.8 mm isotropic). Second, angiograms from the 27 normotensive participants from our previous study^8^ were acquired during free breathing at 3T (GE HDx, Milwaukee, WI; repetition time/echo time=24/2.7 ms, flip angle=20 degrees, voxel size=0.34×0.34×0.5 mm^3^, field of view=192×192×85 mm^3^).

#### Phase-Contrast MR Angiography

This was completed in the hypertensive group only to measure CBF. ECG-gated phase-contrast MR angiography was used to measure CBF in both the left and right vertebral arteries and the common carotid arteries (CCA) at the level of the midneck before the CCA bifurcation where the vessels are perpendicular to the transverse imaging plane. All flow imaging was performed at isocenter.

#### Cardiac MRI

This was performed only in the hypertensive group to measure cardiac output. Short-axis steady-state free precession cines with whole left ventricular coverage (8 mm slice thickness, no slice gap, temporal resolution 38.1 ms, echo time 1.07 ms, in-plane pixel size 1.5×0.8 mm) were used for the estimation of left ventricular volumes, as previously described.^12^ In accordance with the Society of Cardiac Magnetic Resonance guidelines,^13^ a validated^14^ threshold-detection software package was used (cmr42, Circle Cardiovascular Imaging, Canada). Cardiac output was calculated using the left ventricular volumes measured.

### MRI Analysis

#### MRA Analysis

**Figure 1.**
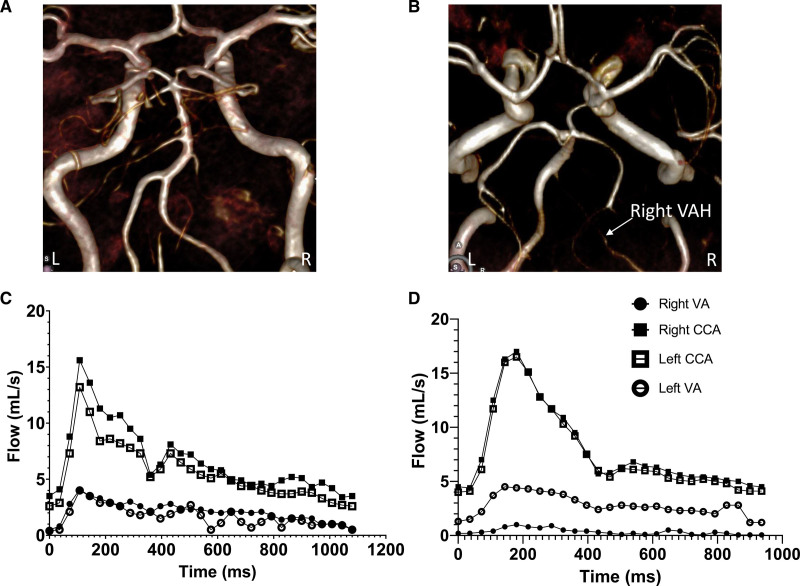
**Three-dimensional MRA reconstructions showing (A) normal caliber vertebral arteries (ie, without hypoplasia) and (B) right vertebral artery (VA) hypoplasia (VAH). C** and **D**, Blood flow throughout the cardiac cycle measured by phase-contrast angiography in the same individuals pictured in (**A**) and (**B**). Data shown is blood flow at each trigger point in the right (R) and left (L) VA and common carotid arteries (CCA) in (**C**) normal caliber VA and (**D**) right VAH.

See Supplemental Material for MRA analysis. Figure 1A and 1B shows examples of normal caliber vertebral arteries and right VAH classified, as previously described.^8,15,16^

#### Phase-Contrast Analysis

CBF data were available in 146 of the 164 young-onset hypertensive patients. Cardiac output was not available in 5 of the patients with total CBF measurements due to technical issues with cardiac cinematic volumetric MR acquisition. The arteries (vertebral and common carotid) were contoured in each frame of reconstructed flow from the smoothed magnitude images. Mean flow velocity and total flow were quantified in each vessel using semi-automated Siemens software (cmr42, Circle Cardiovascular Imaging Solutions). Total CBF was calculated by summing the flow in the vertebral and the common carotid arteries. CBF was indexed for cardiac output since cardiac output influences total CBF^17^ (CBF[L/min]/cardiac output [L/min]×100).

### Statistics

An unpaired Student *t* test was used to test for differences in participant characteristics/demographics between normotensive and hypertensive groups. Binary logistic regression (enter method) with BMI, sex, and age as a covariates was used to test for differences in the prevalence of anatomic variations between hypertensive and normotensive groups. Individuals with normal caliber bilateral vertebral arteries and present or normal caliber bilateral posterior communicating arteries were assigned to a group called normal anatomy. Other people were assigned to an ipCoW only (ipCoW with normal caliber vertebral arteries), VAH only (VAH with normal caliber/present posterior communicating arteries), and VAH+ipCoW. In the young-onset hypertensives, 1-way and 2-way ANOVA’s were used to test for group differences based on anatomical variant in CBF, vertebral artery, or common carotid artery blood flow. All statistical tests were 2-way. Data are presented as mean±SD, median with CI, or mean difference and CI where relevant. Alpha was set at 0.05. Data were analyzed using SPSS (v24, IBM) and Graphpad Prism (v8.4.3).

## Results

### Participants

Table 1 shows participant characteristics and medications. On average, the normotensive (n=56) group was 3 years younger than the hypertensive group (n=164; *P*=0.017), and there were 20% less women in the hypertensive group (*P*=0.013). BMI and body mass were lower in the normotensive group (*P*<0.001 for both). Clinic BP, day-time/nighttime ambulatory BP and were higher in the hypertensive compared with the normotensive group (*P*<0.001).

**Table 1.**
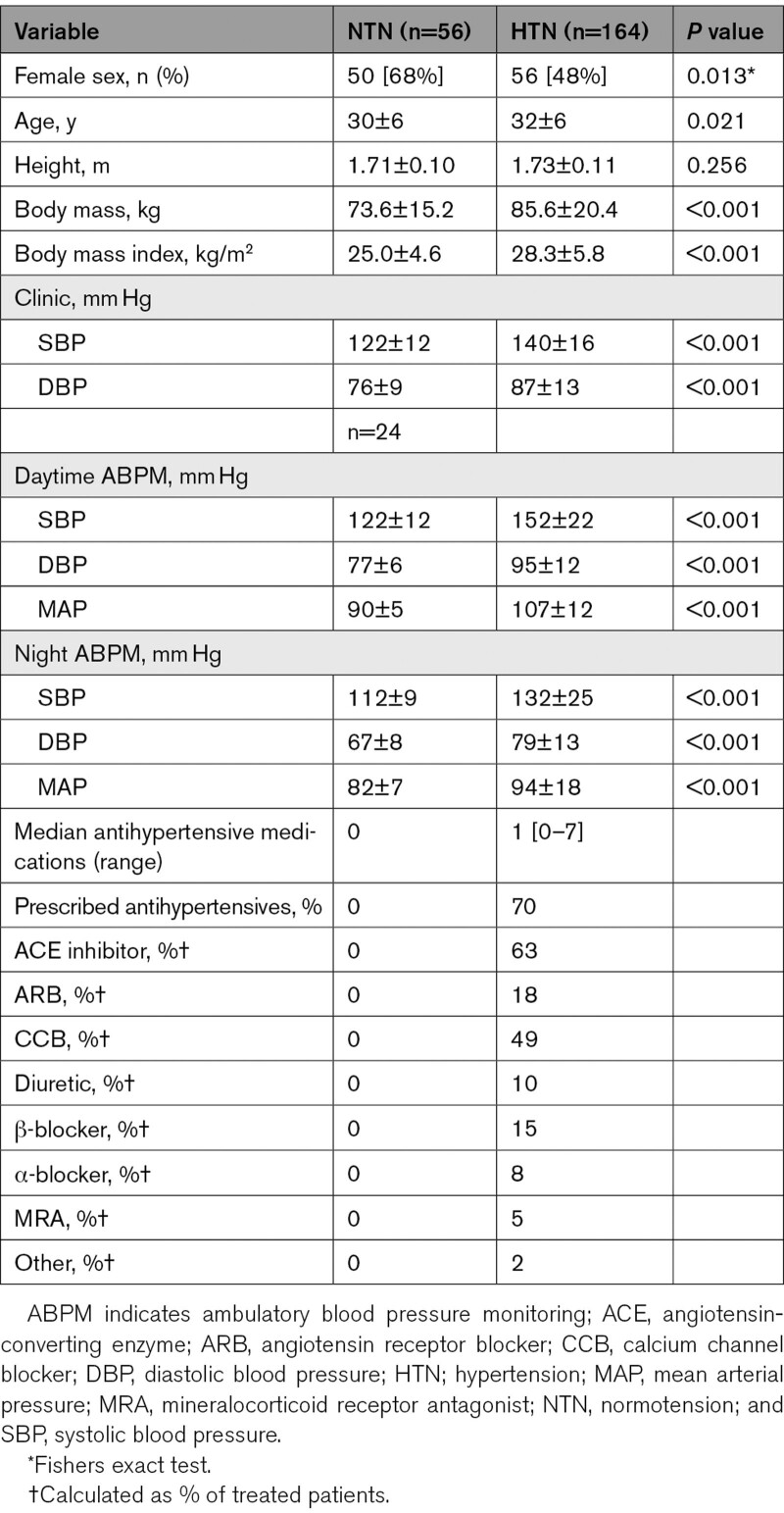
Demographics of Normotensive and Hypertensive Groups

### Are Cerebrovascular Variants Associated With Hypertension?

Table 2 shows the proportions of posterior cerebral circulation variants in both the groups. There was an association of variant type to group, indicating that BP diagnosis was linked to the type of variant classified (Pearson χ^2^ test, *P*<0.001). Binary logistic linear regression with variant type (ie, 4 groups; normal anatomy, ipCoW, VAH, or VAH+ipCoW) as the independent variable and BMI, age, and sex as additional covariates was completed to examine the association between variants and hypertension. Variant type and sex were categorical variables with normal anatomy and male set as the baseline dummy variables respectively. The normal anatomy is defined as bilateral vertebral arteries present or nonhypoplastic and posterior communicating arteries present or nonhypoplastic. VAH+ipCoW was associated with hypertension (odds ratio, 11.79 [95% CI, 3.34–41.58]; *P*<0.001). VAH only (odds ratio, 1.83 [95% CI, 0.56–5.97]; *P*=0.318) and ipCoW only (odds ratio, 1.83 [95% CI, 0.48–2.95]; *P*=0.717) were not associated with hypertension. This means that a person who has VAH+ipCoW has odds of hypertension diagnosis that are 12× that of a person who has a normal anatomy. Additionally, a person with VAH only or ipCoW only is no more likely than a person with the normal anatomy to have hypertension. BMI was associated with hypertension (odds ratio, 1.10 [95% CI, 1.03–1.19]; *P*=0.008). Age (*P*=0.445) and sex (*P*=0.240) were not associated with hypertension in this young cohort.

**Table 2.**
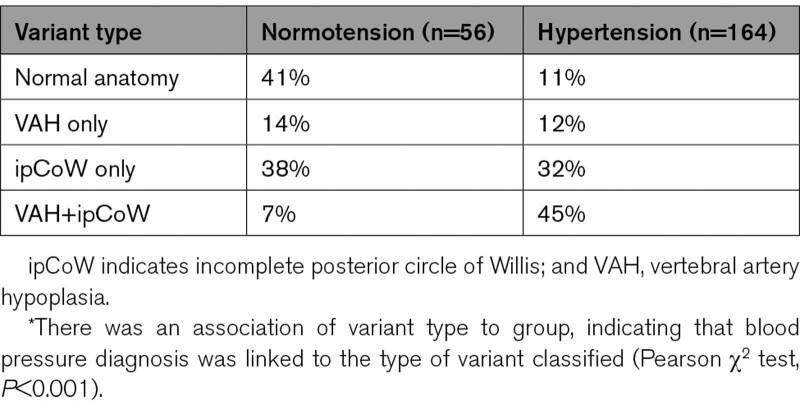
Proportions of Variant Type in the Groups With Normotension and Hypertension*

### Cerebrovascular Variants and Total CBF

To examine whether the identified variants in the posterior cerebral circulation were associated with lower CBF in the young hypertensive patients, we split the group into subgroups classified by their cerebral anatomy. There was no difference in BMI (F=0.98, *P*=0.4038), age (F=1.15, *P*=0.329), clinic systolic BP (F=0.99, *P*=0.3972), clinic diastolic BP (F=0.236, *P*=0.8635), or cardiac output (F=1.75, *P*=0.159) among the participants grouped by variant anatomy (Table 3). There was no association of sex to cerebral vessel anatomy classification in the young-onset hypertensive group (Pearson χ^2^, *P*=0.342, Table 3).

**Table 3.**
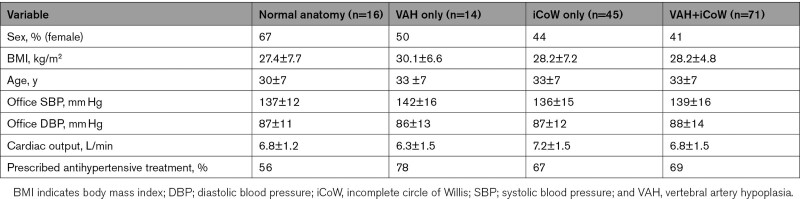
Demographics in Young Hypertensive Patients Grouped by Variant

**Figure 2.**
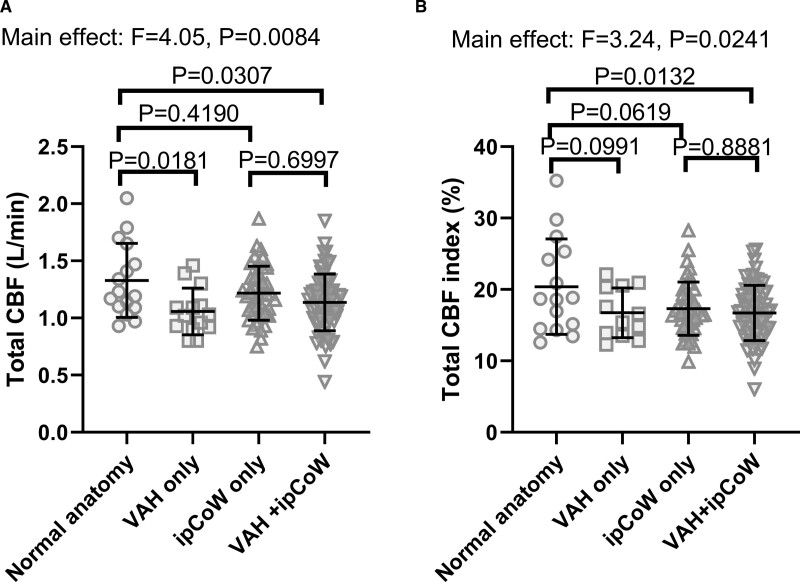
**Absolute total cerebral blood flow (CBF) and CBF indexed for cardiac output in young people with hypertension grouped by cerebral anatomy type. A**, Total CBF in young patients with hypertension and normal posterior variant (ie, no vertebral artery hypoplasia and a complete circle of Willis (CoW), n=16, mean CBF±SD; 1.33±0.32 L/min), incomplete posterior CoW (ipCoW only, n=45, 1.22±0.24 L/min), vertebral artery hypoplasia (VAH only, n=14, 1.06±0.20) and VAH+incomplete circle of Willis (iCoW; n=71, 1.14±0.25 L/min). **B**, Since CBF depends on cardiac output, total CBF was indexed for cardiac output in young patients with hypertension and normal posterior variant (i.e. no vertebral artery hypoplasia and a CoW, n=15, mean±SD; 20.4±6.7%), ipCoW (n=45, 17.3±3.7%), VAH (n=12, 16.7±3.5%) and VAH+iCoW (n=69, 16.7±3.9%). Data are mean±SD. Group means are compared using a 1-way ANOVA with a Tukey test for multiple comparisons. See Tables S1 and S2 for all comparisons.

There was a main effect of anatomical variant on total CBF (*P*=0.008; F=4.054; 1-way ANOVA; Figure 2A for mean±SD). CBF was lower in young hypertension patients with either VAH (n=14, mean difference, −0.27 L/min [95% CI, −0.03 to −0.51 L/min]; *P*=0.0181) or VAH+ipCoW (n=71, mean difference, −0.19 L/min [95% CI, −0.02 to −0.37 L/min]; *P*=0.0307) compared with the normal anatomy group (n=16, Tukey multiple comparisons test). There was no difference in total CBF between groups with ipCoW only (n=45) and normal anatomy (mean difference, −0.12 L/min [95% CI, −0.30 to 0.08 L/min]; *P*=0.4190). See Table S1 for all comparisons.

To check whether the differences in total CBF among hypertensive patients classified by variant type were a result of differences in cardiac output, we indexed total CBF for cardiac output. A main effect of anatomical variant on total CBF indexed for cardiac output persisted (Figure 2B; F=3.24, *P*=0.0241). Indexed total CBF in the VAH+ipCoW group (n=69) was lower compared with the group with the normal anatomy (n=15, mean difference, −3.47% [95% CI, −0.45% to −6.49%]; *P*=0.0172). However, there was no difference in indexed total CBF between groups with VAH only (n=12) and normal anatomy (mean difference, −3.65% [95% CI, −7.86% to 0.55%]; *P*=0.0991; Tukey multiple comparisons test). There was also no difference in indexed total CBF between groups with ipCoW only (n=45) and normal anatomy (mean difference, −3.09% [95% CI, −6.32% to −0.15%]; *P*=0.0679). See Table S2 for all comparisons. Consequently, when total CBF was indexed by cardiac output, CBF was only lower in the group with VAH+ipCoW versus the normal anatomy group. None of the other variants had an effect on total CBF indexed by cardiac output.

### Blood Flow in the Vertebral and Carotid Arteries

**Figure 3.**
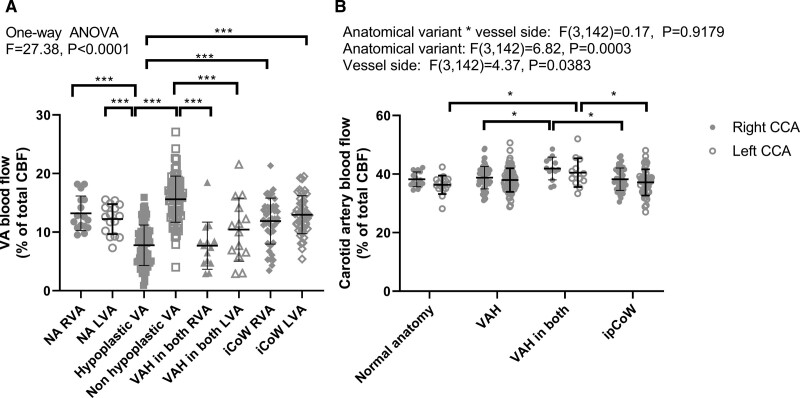
**Blood flow in the vertebral arteries (VA) and carotid arteries (CA) in young people with hypertension grouped by anatomy of the cerebral circulation. A**, Blood flow in VA in patients with anatomy classified as normal anatomy (NA), VA hypoplasia (VAH), and an incomplete circle of Willis (iCoW). Patients were grouped into whether they had VAH in one vessel or both vessels (VAH both). Blood flow in patients with one hypoplastic VA was compared to blood flow in the contralateral vessel and blood flow in the right (R) and left (L) VA in all other groups. VAH in both RVA or LVA means blood flow in the right VA or left VA of patients with VAH in both vessels. Data were compared using a 1-way ANOVA with a Tukey test for multiple comparisons. *P<0.05, ****P<0.0001. See main text for exact *P* values and Table S4A and S4B for all statistical comparisons and descriptive statistics. **B**, Blood flow in the right and left CA in patients classified by cerebral anatomy. Data were compared with a 2-way ANOVA to examine blood flow differences between vessel sides and blood flow difference among the groups. There was no effect of group or vessel side on blood flow in carotid arteries thus no multiple comparisons were completed. See Table S5 for all comparisons and descriptive statistics. Data are mean±SD. CCA indicates common carotid arteries; and ipCoW, incomplete posterior circle of Willis.

We measured whether the variants had a specific impact on blood flow in the vertebral arteries. There was a main effect of cerebral circulation variant on blood flow in the vertebral arteries (1-way ANOVA; F=21.1, *P*<0.0001). Figure 3A shows that blood flow in hypoplastic vertebral arteries is lower versus the normal caliber contralateral vessel (Tukey multiple comparisons test, *P*<0.0001). Blood flow in the hypoplastic vessel was also lower compared to blood flow in the right (*P*<0.0001) and left (*P*<0.0001) vertebral arteries of hypertensives with a normal anatomy. Patients with ipCoW only had a similar blood flow in the right (*P*=0.8113) and left (*P*=0.6836) vertebral arteries compared to patients classified with normal anatomy indicating that hypoplastic or absent posterior communicating arteries did not impact downstream blood flow in the normal caliber vertebral arteries. Patients with hypoplasia in both the right and left vertebral artery had lower blood flow in both vessels versus the right and left vertebral arteries in patients with normal anatomy (*P*=0.0001 and *P*=0.0023, respectively) and patients with ipCoW (*P*=0.0005 and *P*=0.0251, respectively). Table S3A and S3B shows all comparisons and associated statistical variables.

Data were similar when presented as % of total CBF in the left and right vertebral arteries (1-way ANOVA; *P*<0.0001; Figure S2 and Table S4A and S4B for all multiple comparisons). Interestingly, in people with VAH in one vertebral artery, there was a higher % of total blood flow in the nonhypoplastic vessel versus the left vertebral artery of the people with ‘normal’ anatomy (mean difference, 3.39% [95% CI, 0.26 to 6.53]; *P*=0.0237). This may indicate that the contralateral normal caliber vessel tries to correct for the lower blood flow in the hypoplastic vessel.

We then measured whether posterior anatomical variants in the cerebral circulation had a specific impact on blood flow in the carotid arteries. There was no main effect of the vessel side (right or left) or anatomical variant on carotid artery blood flow (2-way ANOVA; F[1,126]=1.1978, *P*=0.1620, Figure 3B). There was no main effect of anatomical variant on right or left carotid artery blood flow (F[3,126]=1.182, *P*=0.3912) indicating that carotid artery blood flow was not different among groups. Table S5 shows descriptive statistics.

When data were analyzed as % of total CBF in the left and right carotid arteries, 2-way ANOVA showed main effect of anatomical variant (F[3,142]=6.82, *P*=0.0003) and vessel side (F[3,1420]=4.373, *P*=0.0383; Figure S2 and Table S5), but there was no interaction between anatomical variant and vessel side (F[3,142]=0.17, *P*=0.9179). Multiple comparisons (Tukey test) showed that people with VAH in one artery only had a lower % of total CBF in the right CCA versus that in people with VAH in both arteries (mean difference, 3.14 [95% CI, −6.21% to −0.07 %]; *P*=0.0433). Also, people with VAH in both arteries had a higher % of CBF in the right CCA versus those with ipCoW only (mean difference, 3.65 [95% CI, 0.45%–6.86%]; *P*=0.0184). In the left CCA, people with VAH in both arteries had a higher % of the total CBF versus people classified with normal anatomy (mean difference, 4.13 [95% CI, 0.32%–7.93%]; *P*=0.0276) and versus people classified with ipCoW only (mean difference, 3.31 [95% CI, 0.10%–6.52%]; *P*=0.0403). These data suggest that hypertensive patients with VAH on both sides have a higher % of total CBF in the CCA, perhaps trying to compensate for lower posterior flow. See Table S6A and S6B for all comparisons and statistics.

## Discussion

The main findings were that (1) VAH+ipCoW was an independently associated with hypertension in adults younger than 40 years old when adjusted for age, BMI, and sex and (2) in young adults with hypertension VAH+ipCoW was associated with lower total CBF.

The higher prevalence of VAH+ipCoW in the young-onset hypertension population (mean age 32±6 years) is similar to our previous findings where we show that this variant was linked to high BP in a population with midlife hypertension (mean age ±SD, 57±2 years)^8^ and in patients with repaired coarctation of the aorta and persistent hypertension (34±14 years).^9^ The percentage of patients with VAH+ipCoW in this study (45%) was similar to that in adults with midlife hypertension (42%).^8^ These findings suggest that the variants are not acquired with age and the uniformly narrow vessels are not likely to be caused by long-term exposure to high BP. We predict that VAH+ipCoW has a causal association with hypertension. However, further work is needed in the form of longitudinal studies. Potentially, young people with normotension and VAH+ipCoW are more likely to develop hypertension in the future.

Exactly why VAH and ipCoW occur in humans is unclear. Data indicate that gestational age is linked to cerebral vessel tortuosity.^18,19^ Potential exposure to different factors in utero and gestational age could cause hypoplastic vessels or cause the lack of development of posterior communicating arteries.

### Why Are Posterior Cerebral Anatomical Variants Associated With Hypertension?

Previously, we showed that posterior variant, VAH, and VAH+ipCoW, were associated with lower CBF in a population of midlife hypertensive patients.^8^ We hypothesized that these variants could cause hypertension by triggering Cushing response, also known as the selfish brain hypothesis, where decreased cerebral perfusion triggers elevated sympathetic nerve activity and BP in order to maintain perfusion.^5,20–22^ In the current study, we show that even in young people with hypertension, VAH+ipCoW was associated with lower cerebral perfusion versus those without these variants, and this was persistent when CBF was indexed for cardiac output. The reduced total CBF was driven by a reduction in blood flow in the vertebral arteries. The lower CBF in patients with VAH+ipCoW occurred despite a large proportion of patients prescribed antihypertensive treatment (69%), suggesting that treatment may not protect even young patients with VAH+ipCoW against a lower CBF. However, further work needs to be completed examining whether specific classes of antihypertensive medications may help to improve perfusion or prevent a decline in perfusion with age in a group of hypertensive young people with VAH plus ipCoW. In the SMART MR study (patients with midlife hypertension and manifest atherosclerotic disease) patients prescribed angiotensin receptor blockers did not show a decline CBF over a period of 3.9 years, whereas CBF declined in patients taking other classes of antihypertensive medications (calcium channel blockers, angiotensin-converting enzyme inhibitors, diuretics, and beta-blockers).^23^

We examined whether VAH alone, ipCoW alone, or VAH with ipCoW had more impact on CBF. We found that patients with ipCoW had a similar CBF (absolute or % of cardiac output) to patients with normal caliber posterior communicating arteries suggesting that either missing or hypoplastic posterior communicating arteries does not impact total CBF. The caveat of this analysis is that we were not measuring regional CBF, and this variant may impact blood flow to posterior territories. However, we did find that VAH alone was associated with reduced total CBF versus that in the group classified with normal anatomy; however, this difference was not evident when we indexed CBF for cardiac output, indicating that it is VAH+ipCoW that has the greatest impact on CBF.

Since VAH+ipCoW impacts CBF even in young adults with hypertension, potentially patients with this combined variant are more likely to develop cerebrovascular disease earlier on in life versus patients who do not have this variant. Along these lines, Suvila et al^24^ showed that early-onset hypertension was associated with cognitive impairment, whereas late-onset hypertension was not. Potentially, young adults with VAH+ipCoW and hypertension are more at risk of cognitive decline earlier on in life. Unfortunately, we do not have measurements of CBF in the normotensive group to compare to our hypertensive group. Currently, it is unknown whether hypertension is linked to lower CBF in young adults with hypertension, as it is in people with midlife hypertension.^8,23^ Williamson et al^18^ showed that in young adults (≈age 24 years) without cerebrovascular disease, a poor cardiovascular risk score (including high systolic BP) was associated with lower CBF.

Exactly how lower cerebral perfusion could trigger elevated BP in humans is unknown. Recently, Marina et al^25^ showed that in rats, astrocytes can detect decreasing CBF and activate the sympathetic nervous system to increase BP in order to maintain perfusion. It is possible that with age, there is a larger decline in CBF in people with VAH and astrocytes could be the intracranial perfusion monitors via which hypertension develops in some patients.

### Other Mechanisms

VAH with ipCoW may be related to hypertension via common factors/mechanisms (e.g. genes). Potentially, VAH and ipCoW signposts a developmental issue in the global vasculature or certain areas of the vasculature. For example, VAH could represent generally stiffer vessels due to higher collagen content. It could be that single nucleotide polymorphisms in certain genes that regulate vessel development, causing VAH, also cause vascular dysfunction, arterial stiffness, and hypertension. In our previous study, we found that anterior cerebrovascular variants were not associated with hypertension,^8^ suggesting that posterior anatomical variants are specifically associated with developing high BP.

### Limitations

The control group was a combination of data taken from a previous study by our group^8^ who were screened before participating, and data taken from MR angiograms on individuals with pulsatile tinnitus for which no known cause was found and in whom had otherwise no significant intracerebral abnormality. The latter group of participants were identified as normotensive by medical records and office BP. However, ambulatory BP was not completed to confirm BP status. Thus the control group may not represent the normotensive population at large. Additionally, this study was a retrospective cross-sectional study and thus can only show an association. Whether VAH+ipCoW is causal in hypertension and reduces overall cerebral perfusion remains to be confirmed via prospective longitudinal studies. Finally, we cannot rule out that the variants reported here are acquired rather than congenital. It is possible that raised BP in adolescence could have led to acquired vascular abnormalities and vessel thickening in the patients reported here. However, acquired vascular stenoses and narrowing typically do not present as uniform diffusely small vessels without wall thickening, which is what we are reporting as hypoplasia. The patients included did not have evidence of stenoses or focal narrowing anywhere in cerebrovascular circulation

### Perspectives

Our data indicate that variants in the anatomy of the posterior circulation, specifically, the combination of VAH and an incomplete posterior CoW are far more common in people with young-onset hypertension. This is consistent with previous studies in people with midlife hypertension, indicating that these variants are not acquired with aging into midlife and could be causally associated with the onset of hypertension. We show that posterior cerebrovascular variants are associated with lower total CBF in young patients with hypertension. Potentially VAH+ipCoW may specifically place these patients at risk of developing cerebrovascular disease earlier on in life versus patients who do not have this variant. Understanding how to treat young patients with hypertension and posterior cerebrovascular variants could improve their long-term outlook for cerebrovascular pathologies. For example, should these patients be treated earlier (before evidence of end-organ damage appears) or is there a specific antihypertensive medication that may improve not only BP but also CBF in young patients with VAH+ipCoW? More research is needed to understand the long-term consequences of VAH+ipCoW on cardiovascular and cerebrovascular health.

## Article Information

### Sources of Funding

British Heart Foundation; FS/18/18/33522 (T. Hinton), AA/18/7/34219 (E.C. Hart), FS/11/1/28400 (E.C. Hart), and Health Research Council of New Zealand, Sidney Taylor Trust (J.F.R. Paton).

### Disclosures

None.

## Supplementary Material



## References

[1] Collaborators GRF. Global, regional, and national comparative risk assessment of 84 behavioural, environmental and occupational, and metabolic risks or clusters of risks for 195 countries and territories, 1990-2017: a systematic analysis for the Global Burden of Disease Study 2017. Lancet. 2018;392:1923–1994. doi: 10.1016/S0140-6736(18)32225-6 30496105 10.1016/S0140-6736(18)32225-6 PMC6227755

[2] Kearney PM Whelton M Reynolds K Muntner P Whelton PK He J. Global burden of hypertension: analysis of worldwide data. Lancet. 2005;365:217–223. doi: 10.1016/S0140-6736(05)17741-1 15652604 10.1016/S0140-6736(05)17741-1

[3] Yano Y Reis JP Colangelo LA Shimbo D Viera AJ Allen NB Gidding SS Bress AP Greenland P Muntner P. Association of blood pressure classification in young adults using the 2017 American College of Cardiology/American Heart Association blood pressure guideline with cardiovascular events later in life. JAMA. 2018;320:1774–1782. doi: 10.1001/jama.2018.13551 30398601 10.1001/jama.2018.13551 PMC6248102

[4] Grassi G Mark A Esler M. The sympathetic nervous system alterations in human hypertension. Circ Res. 2015;116:976–990. doi: 10.1161/CIRCRESAHA.116.303604 25767284 10.1161/CIRCRESAHA.116.303604 PMC4367954

[5] Cates MJ Dickinson CJ Hart EC Paton JF. Neurogenic hypertension and elevated vertebrobasilar arterial resistance: is there a causative link? Curr Hypertens Rep. 2012;14:261–269. doi: 10.1007/s11906-012-0267-6 22562144 10.1007/s11906-012-0267-6

[6] Cates MJ Steed PW Abdala AP Langton PD Paton JF. Elevated vertebrobasilar artery resistance in neonatal spontaneously hypertensive rats. J Appl Physiol (1985). 2011;111:149–156. doi: 10.1152/japplphysiol.00220.2011 21493719 10.1152/japplphysiol.00220.2011 PMC3137540

[7] Waki H Bhuiyan ME Gouraud SS Takagishi M Hatada A Kohsaka A Paton JF Maeda M. Acute reductions in blood flow restricted to the dorsomedial medulla induce a pressor response in rats. J Hypertens. 2011;29:1536–1545. doi: 10.1097/HJH.0b013e3283484106 21666494 10.1097/HJH.0b013e3283484106

[8] Warnert EA Rodrigues JC Burchell AE Neumann S Ratcliffe LE Manghat NE Harris AD Adams Z Nightingale AK Wise RG. Is high blood pressure self-protection for the brain? Circ Res. 2016;119:e140–e151. doi: 10.1161/CIRCRESAHA.116.309493 27672161 10.1161/CIRCRESAHA.116.309493

[9] Rodrigues JCL Jaring MFR Werndle MC Mitrousi K Lyen SM Nightingale AK Hamilton MCK Curtis SL Manghat NE Paton JFR. Repaired coarctation of the aorta, persistent arterial hypertension and the selfish brain. J Cardiovasc Magn Reson. 2019;21:68. doi: 10.1186/s12968-019-0578-8 31703697 10.1186/s12968-019-0578-8 PMC6839237

[10] Burchell AE Rodrigues JC Charalambos M Ratcliffe LE Hart EC Paton JF Baumbach A Manghat NE Nightingale AK. Comprehensive first-line magnetic resonance imaging in hypertension: experience from a single-center tertiary referral clinic. J Clin Hypertens (Greenwich). 2017;19:13–22. doi: 10.1111/jch.12920 27759186 10.1111/jch.12920 PMC8031106

[11] Williams B Mancia G Spiering W Agabiti Rosei E Azizi M Burnier M Clement D Coca A De Simone G Dominiczak A. 2018 Practice Guidelines for the management of arterial hypertension of the European Society of Cardiology and the European Society of Hypertension. Blood Press. 2018;27:314–340. doi: 10.1080/08037051.2018.1527177 30380928 10.1080/08037051.2018.1527177

[12] Maceira AM Prasad SK Khan M Pennell DJ. Normalized left ventricular systolic and diastolic function by steady state free precession cardiovascular magnetic resonance. J Cardiovasc Magn Reson. 2006;8:417–426. doi: 10.1080/10976640600572889 16755827 10.1080/10976640600572889

[13] Schulz-Menger J Bluemke DA Bremerich J Flamm SD Fogel MA Friedrich MG Kim RJ von Knobelsdorff-Brenkenhoff F Kramer CM Pennell DJ. Standardized image interpretation and post processing in cardiovascular magnetic resonance: Society for Cardiovascular Magnetic Resonance (SCMR) board of trustees task force on standardized post processing. J Cardiovasc Magn Reson. 2013;15:35. doi: 10.1186/1532-429X-15-35 23634753 10.1186/1532-429X-15-35 PMC3695769

[14] Childs H Ma L Ma M Clarke J Cocker M Green J Strohm O Friedrich MG. Comparison of long and short axis quantification of left ventricular volume parameters by cardiovascular magnetic resonance, with ex-vivo validation. J Cardiovasc Magn Reson. 2011;13:40. doi: 10.1186/1532-429X-13-40 21834992 10.1186/1532-429X-13-40 PMC3169477

[15] Park JH Kim JM Roh JK. Hypoplastic vertebral artery: frequency and associations with ischaemic stroke territory. J Neurol Neurosurg Psychiatry. 2007;78:954–958. doi: 10.1136/jnnp.2006.105767 17098838 10.1136/jnnp.2006.105767 PMC2117863

[16] Krabbe-Hartkamp MJ van der Grond J de Leeuw FE de Groot JC Algra A Hillen B Breteler MM Mali WP. Circle of Willis: morphologic variation on three-dimensional time-of-flight MR angiograms. Radiology. 1998;207:103–111. doi: 10.1148/radiology.207.1.9530305 9530305 10.1148/radiology.207.1.9530305

[17] Neumann S Burchell AE Rodrigues JCL Lawton CB Burden D Underhill M Kobetić MD Adams ZH Brooks JCW Nightingale AK. Cerebral blood flow response to simulated hypovolemia in essential hypertension: a magnetic resonance imaging study. Hypertension. 2019;74:1391–1398. doi: 10.1161/HYPERTENSIONAHA.119.13229 31656098 10.1161/HYPERTENSIONAHA.119.13229 PMC7069391

[18] Williamson W Lewandowski AJ Forkert ND Griffanti L Okell TW Betts J Boardman H Siepmann T McKean D Huckstep O. Association of cardiovascular risk factors with MRI indices of cerebrovascular structure and function and white matter hyperintensities in young adults. JAMA. 2018;320:665–673. doi: 10.1001/jama.2018.11498 30140877 10.1001/jama.2018.11498 PMC6142949

[19] Raikkonen K Kajantie E Pesonen AK Heinonen K Alastalo H Leskinen JT Nyman K Henriksson M Lahti J Lahti M. Early life origins cognitive decline: findings in elderly men in the Helsinki Birth Cohort Study. PLoS One. 2013;8:e54707. doi: 10.1371/journal.pone.0054707 23382945 10.1371/journal.pone.0054707 PMC3559835

[20] Guild SJ Saxena UA McBryde FD Malpas SC Ramchandra R. Intracranial pressure influences the level of sympathetic tone. Am J Physiol Regul Integr Comp Physiol. 2018;315:R1049–R1053. doi: 10.1152/ajpregu.00183.2018 30207755 10.1152/ajpregu.00183.2018

[21] Schmidt EA Despas F Pavy-Le Traon A Czosnyka Z Pickard JD Rahmouni K Pathak A Senard JM. Intracranial pressure is a determinant of sympathetic activity. Front Physiol. 2018;9:11. doi: 10.3389/fphys.2018.00011 29472865 10.3389/fphys.2018.00011 PMC5809772

[22] Cushing H. Concerning a definitive regulatory mechanism of the vasomotor centre which controls blood pressure during cerebral compression. Bull Johns Hopk Hosp. 1901:290–292.

[23] Muller M van der Graaf Y Visseren FL Mali WP Geerlings MI; SMART Study Group. Hypertension and longitudinal changes in cerebral blood flow: the SMART-MR study. Ann Neurol. 2012;71:825–833. doi: 10.1002/ana.23554 22447734 10.1002/ana.23554

[24] Suvila K Lima JAC Yano Y Tan ZS Cheng S Niiranen TJ. Early-but not late-onset hypertension is related to midlife cognitive function. Hypertension. 2021;77:972–979. doi: 10.1161/HYPERTENSIONAHA.120.16556 33461314 10.1161/HYPERTENSIONAHA.120.16556 PMC7878356

[25] Marina N Christie IN Korsak A Doronin M Brazhe A Hosford PS Wells JA Sheikhbahaei S Humoud I Paton JFR. Astrocytes monitor cerebral perfusion and control systemic circulation to maintain brain blood flow. Nat Commun. 2020;11:131. doi: 10.1038/s41467-019-13956-y 31919423 10.1038/s41467-019-13956-y PMC6952443

