# Supplementary material for: Cerebrovascular Variants and the Role of the Selfish Brain in Young-Onset Hypertension
Source: Hypertension. 2022 Mar 16;79(6):1265–74. doi: 10.1161/HYPERTENSIONAHA.121.18612 (PMC9093235; doi:10.1161/HYPERTENSIONAHA.121.18612)
Supplement: Supplementary file 1 [file hyp-79-1265-s001.pdf]

Cerebrovascular variants and the role of the selfish brain in young onset hypertension

Nathan E. Manghat*[1,2] Elizabeth Robinson*[1], Konstantina Mitrousi*[1,2], Jonathan C. L. Rodrigues[3,4], Thomas Hinton[2], Julian F.R. Paton[5], Richard G. Wise[6,7,8], Angus K. Nightingale[2,9] and Emma C Hart[2,9]

* these authors contributed equally to first authorship

[1]Department of Radiology, Bristol Royal Infirmary, University Hospitals Bristol and Weston NHS Foundation Trust, UK.

[2]Cardionomics Research Group, School of Physiology, Pharmacology and Neurosciences, University of Bristol, UK.

[3]Department of Radiology, Royal United Hospitals Bath NHS Foundation Trust

[4] Department of Health, University of Bath, UK.

[5] Manaaki Manawa, The Centre for Heart Research, Department of Physiology, Faculty of Medical & Health Sciences, University of Auckland, Auckland 1142, New Zealand

[6] Cardiff University Brain Research Imaging Centre, School of Psychology, Cardiff University,

[7] Department of Neuroscience, Imaging and Clinical Sciences, "G. D'Annunzio" University of Chieti-Pescara, Italy.

[8]ITAB-Institute for Advanced Biomedical Technologies, "G. D'Annunzio" University of Chieti-Pescara, Chieti, Italy.

[9]Clinical Research Facility, University Hospitals Bristol and Weston NHS Foundation Trust, UK.

Supplementary material

Supplemental methods:

MRA analysis: MRA data were reviewed in 3 orthogonal multiplanar reformatted (MPR) planes with cross-referencing of images. Maximum intensity projection images were generated and reviewed. Scans were routinely reported by a consultant cardiovascular radiologist with over 12 years of experience and retrospectively independently reviewed by a radiologist with > 3 years' experience blinded to clinical details, including normotensive/hypertensive state. Discrepancies were resolved by consensus. All MRAs were reviewed on dedicated workstations (Insignia Medical Systems, United Kingdom). The visualised V2 (portion in the vertebral columns), V3 (after exit from the C2 transverse foramen) and V4 (the intracranial portion beginning at the atlanto-occipital membrane and terminating at the basilar artery) segments were analysed. VAH was defined as a diameter < 2 mm uniformly throughout the vessel or diffusely reduced calibre of the V4 segment, and not if only a focal narrowing was presented suggestive of atherosclerotic steno-occlusive disease[15] . CoW anatomy was classified as previously described [16]. Briefly, vessels that were visualized as continuous segments of at least 0.8 mm in diameter were considered present and those smaller than 0.8 mm in diameter were considered hypoplastic [16]. These predefined calibre thresholds facilitated direct comparison between 1.5 T and higher resolution 3 T MRA datasets. Care was taken to distinguish the posterior communicating arteries from the anterior choroidal arteries by cross-referencing MPR images. The connection of the posterior communicating artery with the posterior cerebral artery was confirmed for all posterior communicating arteries identified. The posterior aspect of each CoW was assessed for morphology and classified as previously described [16]. Incomplete posterior CoW (ipCoW) was defined as either unilateral or bilateral hypoplastic or absent posterior communicating arteries or unilateral or bilateral hypoplasia.

Tables

Table S1: Ordinary one-way ANOVA and Tukey's multiple comparison test data for the effect of variant type on cerebral blood flow. Sample size in each group are: normal anatomy; n=16, ipCoW; n=45, VAH+ipCoW; n=71 and VAH only; n=14)

Comparisons	Mean difference (l/min)	95% CI of the difference (l/min)	Adjusted P-value
Normal anatomy vs. VAH only	0.27	0.03 to 0.51	0.0181
Normal anatomy vs. ipCoW only	0.11	-0.08 to 0.30	0.4190
Normal variant vs. VAH +ipCoW	0.19	0.01 to 0.37	0.0307
VAH only vs. ipCoW only	-0.16	-0.36 to 0.04	0.1604
VAH only vs. VAH +ipCoW	-0.08	-0.27 to 0.11	0.6997
ipCoW only vs. VAH +ipCoW	0.08	-0.04 to 0.20	0.3280

VAH; vertebral artery hypoplasia, ipCoW; incomplete posterior circle of Willis

Table S2: Ordinary one-way ANOVA and Tukey's multiple comparison test data for the effect of variant type on cerebral blood flow indexed for cardiac output. Sample size in each group are: normal anatomy; n=15, ipCoW; n=45, VAH+ipCoW; n=69 and VAH only; n=12)

Comparisons	Mean difference (% of cardiac output)	95% CI of the difference (% of cardiac output)	Adjusted P-value
Normal anatomy vs. VAH only	3.652	-0.5549 to 7.858	0.1132
Normal anatomy vs. ipCoW only	3.085	-0.1530 to 6.324	0.0679
Normal anatomy vs. VAH+ipCoW	3.663	0.5688 to 6.757	0.0132
VAH only vs. ipCoW only	-0.5665	-4.095 to 2.962	0.9754
VAH only vs. VAH+ipCoW	0.01127	-3.386 to 3.408	>0.9999
ipCoW only vs. VAH+ipCoW	0.5778	-1.503 to 2.65	0.8881

Table S3A: Ordinary one-way ANOVA and Tukey's multiple comparison test data for the effect of variant type on left (L) and right (R) vertebral artery (VA) blood flow. Sample sizes for each group are: Normal anatomy (NA); n=16, ipCoW; n=45, Hypoplastic VAH/non hypoplastic VAH; n=71, and VAH in both arteries; n=14. Supplementary Table 3B shows the mean +/-SD

Comparisons	Mean difference (l/min	95% CI of the difference l/min)	Adjusted P-value
NA-RVA vs. NA-LVA	0.007500	-0.04488 to 0.05988	0.9999
NA-RVA vs. VAH	0.09113	0.04944 to 0.1328	<0.0001
NA-RVA vs. Contralateral VA	0.002964	-0.03858 to 0.04451	>0.9999
NA-RVA vs. ipCoW-RVA	0.02041	-0.02135 to 0.06217	0.8113
NA-RVA vs. ipCoW-LVA	0.02329	-0.01840 to 0.06498	0.6836
NA-RVA vs. VAH in both-RVA	0.08000	0.02762 to 0.1324	0.0001
NA-RVA vs. VAH in both-LVA	0.06813	0.01574 to 0.1205	0.0023
NA-LVA vs. VAH	0.08363	0.04194 to 0.1253	<0.0001
NA-LVA vs. Contralateral VA	-0.004536	-0.04608 to 0.03701	>0.9999
NA-LVA vs. ipCoW-RVA	0.01291	-0.02885 to 0.05467	0.9814
NA-LVA vs. ipCoW-LVA	0.01579	-0.02590 to 0.05748	0.9433
NA-LVA vs. VAH in both-RVA	0.07250	0.02012 to 0.1249	0.0008
NA-LVA vs. VAH in both-LVA	0.06063	0.008243 to 0.1130	0.0111
VAH vs. Contralateral VA	-0.08816	-0.1150 to -0.06133	<0.0001
VAH vs. ipCoW-RVA	-0.07071	-0.09788 to -0.04355	<0.0001
VAH vs. ipCoW-LVA	-0.06783	-0.09488 to -0.04078	<0.0001
VAH vs. VAH in both-RVA	-0.01113	-0.05281 to 0.03056	0.9922
VAH vs. VAH in both-LVA	-0.02300	-0.06469 to 0.01869	0.6975
Contralateral VA vs. ipCoW-RVA	0.01745	-0.009497 to 0.04440	0.4994
Contralateral VA vs. ipCoW-LVA	0.02033	-0.006503 to 0.04716	0.2901
Contralateral VA vs. VAH in both-RVA	0.07704	0.03549 to 0.1186	<0.0001
Contralateral VA vs. VAH in both-LVA	0.06516	0.02362 to 0.1067	<0.0001
ipCoW-RVA vs. ipCoW-LVA	0.002879	-0.02429 to 0.03004	>0.9999
ipCoW-RVA vs. VAH in both-RVA	0.05959	0.01783 to 0.1013	0.0005
ipCoW-RVA vs. VAH in both-LVA	0.04771	0.005951 to 0.08947	0.0129
ipCoW-LVA vs. VAH in both-RVA	0.05671	0.01502 to 0.09840	0.0011
ipCoW-LVA vs. VAH in both-LVA	0.04483	0.003146 to 0.08652	0.0251
VAH in both-RVA vs. VAH in both-LVA	-0.01188	-0.06426 to 0.04051	0.9972

Table S3B: Mean and standard deviation (SD) of the total blood flow in a specific vertebral artery in each group of hypertensive patients categorised by variant type.

**Group and vessel**	**Mean (l/min)**	**SD (l/min)**
NA-RVA	0.173	0.049
NA-LVA	0.166	0.043
VAH	0.082	0.046
Contralateral VA	0.170	0.057
ipCoW-RVA	0.153	0.044
ipCoW-LVA	0.150	0.046
VAH in both-RVA	0.093	0.042
VAH in both-LVA	0.105	0.060

Table S4A: Ordinary one-way ANOVA and Tukey's multiple comparison test data for the effect of variant type on left (L) and right (R) vertebral artery (VA) blood flow as a **% of the total CBF**. Sample sizes for each group are: Normal anatomy (NA); n=16, ipCoW; n=45, Hypoplastic VAH/non hypoplastic VAH; n=71, and VAH in both arteries; n=14.

**Tukey's multiple comparisons test**	**Mean difference (l/min)**	**95.00% CI of difference**	**Adjusted P Value**
NA RVA vs. NV LVA	0.9738	-3.040 to 4.987	0.9956
NA RVA vs. ipCoW RVA	1.314	-2.000 to 4.628	0.9282
NA RVA vs. ipCoW LVA	0.2328	-3.071 to 3.537	>0.9999
NA RVA vs. Hypoplastic VA	5.550	2.413 to 8.687	<0.0001
NA RVA vs. Non hypoplastic VA	-2.419	-5.556 to 0.7184	0.2683
NA RVA vs. VAH in both RVA	5.516	1.362 to 9.670	0.0017
NA RVA vs. VAH in both LVA	2.778	-1.377 to 6.932	0.4557
NA LVA vs. ipCoW RVA	0.3405	-2.973 to 3.654	>0.9999
NA LVA vs. ipCoW LVA	-0.7410	-4.045 to 2.563	0.9973
NA LVA vs. Hypoplastic VA	4.576	1.439 to 7.714	0.0003
NA LVA vs. Non hypoplastic VA	-3.393	-6.530 to -0.2554	0.0237
NA LVA vs. VAH in both RVA	4.542	0.3880 to 8.696	0.0211
NA LVA vs. VAH in both LVA	1.804	-2.350 to 5.958	0.8884
ipCoW RVA vs. ipCoW LVA	-1.082	-3.488 to 1.325	0.8692
ipCoW RVA vs. Hypoplastic VA	4.236	2.064 to 6.408	<0.0001
ipCoW RVA vs. Non hypoplastic VA	-3.733	-5.905 to -1.561	<0.0001
ipCoW RVA vs. VAH in both RVA	4.202	0.7186 to 7.685	0.0066
ipCoW RVA vs. VAH in both LVA	1.463	-2.020 to 4.947	0.9046
ipCoW LVA vs. Hypoplastic VA	5.317	3.160 to 7.474	<0.0001
ipCoW LVA vs. Non hypoplastic VA	-2.652	-4.809 to -0.4946	0.0051
ipCoW LVA vs. VAH in both RVA	5.283	1.809 to 8.757	0.0001
ipCoW LVA vs. VAH in both LVA	2.545	-0.9289 to 6.019	0.3329
Hypoplastic VA vs. Non hypoplastic VA	-7.969	-9.861 to -6.077	<0.0001
Hypoplastic VA vs. VAH in both RVA	-0.03404	-3.350 to 3.282	>0.9999
Hypoplastic VA vs. VAH in both LVA	-2.772	-6.088 to 0.5432	0.1779
Non hypoplastic VA vs. VAH in both RVA	7.935	4.619 to 11.25	<0.0001
Non hypoplastic VA vs. VAH in both LVA	5.197	1.881 to 8.512	<0.0001
VAH in both RVA vs. VAH in both LVA	-2.738	-7.029 to 1.552	0.518

Table S4B: Mean and SD of the % of total blood flow in a specific vertebral artery in each group of hypertensive patients categorised by variant type.

**Group and vessel side**	**Mean (%)**	**SD (%)**
NA-RVA	13.20	2.95
NA-LVA	12.23	3.93
Hypoplastic VA	7.66	3.56
Contralateral VA	15.62	3.93
ipCoW-RVA	11.89	3.93
ipCoW-LVA	12.97	3.24
VAH in both-RVA	7.69	4.02
VAH in both-LVA	10.43	5.38

Table S5: Mean and SD for the right or left CCA blood flow in each group of hypertensive patients categorised by variant type.

Group and vessel	Mean (l/min)	SD (l/min
NA-RCCA	0.506	0.123
NA-LCCA	0.488	0.151
VAH on one side-RCCA	0.440	0.132
VAH on one side-LCCA	0.437	0.123
VAH in both-RCCA	0.450	0.103
VAH in both-lCCA	0.440	0.095
ipCoW-RCCA	0.460	0.089
ipCoW-LCCA	0.449	0.106

Table S6A: Mixed effects two way ANOVA and Tukey's multiple comparison test data for the effect of variant type on left (L) and right (R) common carotid artery (CCA) blood flow as a **% of the total CBF**. Sample sizes for each group are: Normal anatomy (NA); n=16, ipCoW; n=45, Hypoplastic VAH/non hypoplastic VAH; n=71, and VAH in both arteries; n=14.

Comparisons	Mean difference (%)	95.00% CI of difference.	Adjusted P Value
**Right CCA**			
Normal anatomy vs. VAH	-0.5382	-3.354 to 2.278	0.9604
Normal anatomy vs. VAH in both	-3.674	-7.479 to 0.1307	0.0627
Normal anatomy vs. ipCoW	-0.02013	-2.986 to 2.946	>0.9999
VAH vs. VAH in both	-3.136	-6.206 to -0.06517	0.0433
VAH vs. ipCoW	0.5181	-1.418 to 2.454	0.9003
VAH in both vs. ipCoW	3.654	0.4455 to 6.862	0.0184
**Left CCA**			
Normal anatomy vs. VAH	-1.619	-4.435 to 1.197	0.4474
Normal anatomy vs. VAH in both	-4.127	-7.932 to -0.3223	0.0276
Normal anatomy vs. ipCoW	-0.8174	-3.783 to 2.149	0.8922
VAH vs. VAH in both	-2.508	-5.578 to 0.5628	0.1522
VAH vs. ipCoW	0.8017	-1.135 to 2.738	0.7081
VAH in both vs. ipCoW	3.310	0.1012 to 6.518	0.0403

Table S6B: Mean and SD of the % of total blood flow in the right or left CCA in each hypertensive group categorised by anatomical variant.

Group and vessel side	Mean (%)	SD (%)
NA-RCCA	38.22	2.53
NA-LCCA	36.35	3.15
VAH on one side-RCCA	38.76	3.82
VAH on one side-LCCA	37.97	4.02
VAH in both-RCCA	41.89	3.86
VAH in both-lCCA	40.47	4.90
ipCoW-RCCA	38.24	3.81
ipCoW-LCCA	37.16	4.48

Supplementary Figures

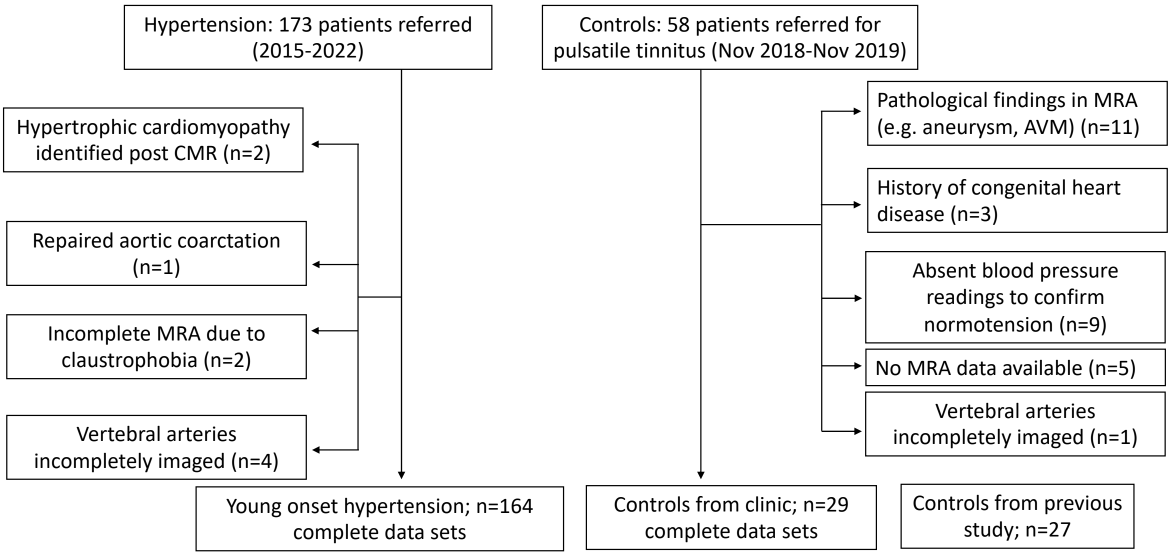

Figure S1: Flow chart showing cases that were identified and excluded, and the final sample size for each group. CMR; cardiac magnetic resonance imaging, MRA; magnetic resonance angiography, AVM; arteriovenous malformation.

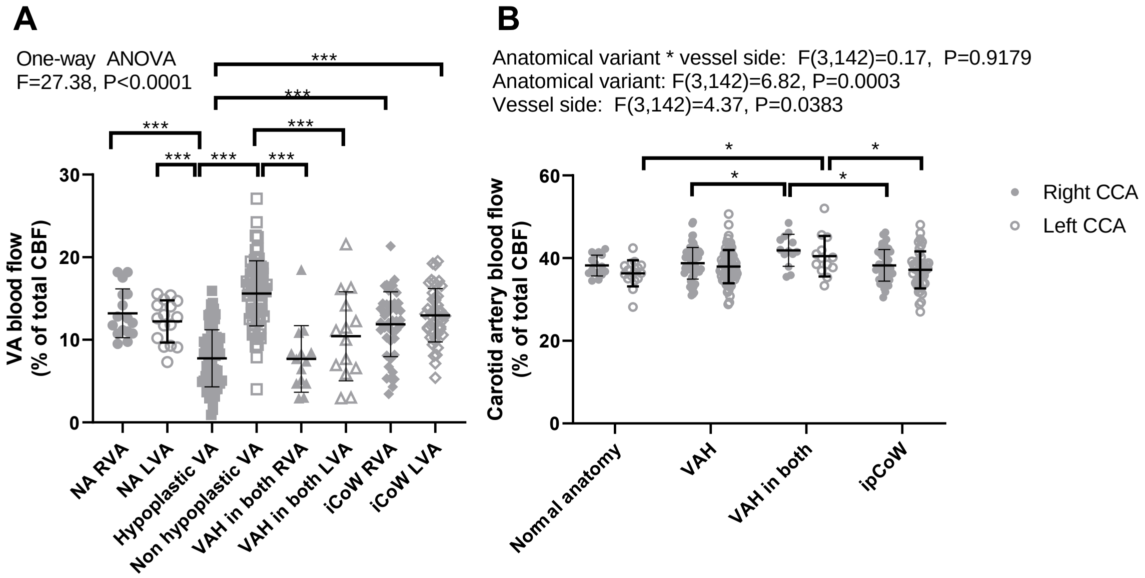

Figure S2: A) Blood flow as a % of total cerebral blood flow (CBF) in the right (R) and left (L) vertebral arteries (VA) in young onset hypertensive patients categorised with 1) normal anatomy (NA, n=16), 2) hypoplastic VA in one artery with their contralateral vessel as a normal calibre VA (non-hypoplastic VA, n=71), 3) vertebral artery hypoplasia in both arteries (VAH in both, n=14) and 4) an incomplete posterior circle of Willis only (ipCoW, n=45). VAH in both RVA or LVA corresponds to blood flow in the right VA or left VA in people with VAH in both vertebral arteries. See supplementary table 4 for multiple comparisons (Tukey tests) and adjusted P-values. B) Blood flow as a % of total CBF in the right (R) and left (L) common carotid arteries (CCA) in young onset hypertensive patients categorised with 1) normal anatomy, 2) VAH in one vessel, 3) VAH in both arteries and 4) ipCoW only. See supplementary table 5 for multiple comparisons and adjusted P-values.
